# Neodymium magnet-assisted retrieval of metallic foreign bodies in the upper extremity: a descriptive surgical technique series

**DOI:** 10.3389/fsurg.2026.1854795

**Published:** 2026-07-15

**Authors:** Okyar Altas, Kemal Zencirli, Kagan Cevlik, Dogan Kiral, Alperen Korucu, Alperen Elibol

**Affiliations:** 1 Department of Hand Surgery, Başakşehir Çam and Sakura City Hospital, Istanbul, Türkiye; 2 Department of Orthopaedics and Traumatology, Gazi Yaşargil Education and Research Hospital, Diyarbakır, Türkiye; 3 Korucu Clinic, Istanbul, Turkey; 4 Halic University, Istanbul, Turkey

**Keywords:** ferromagnetic retrieval, hand surgery, metallic foreign body, minimally invasive surgery, neodymium magnets, surgical technique, upper extremity

## Abstract

**Background:**

Retained metallic foreign bodies (MFBs) of the upper extremity constitute a clinically consequential subset of penetrating soft tissue injuries. Conventional retrieval approaches may necessitate extensive tissue dissection, prolonged operative durations, and repeated intraoperative fluoroscopic imaging. Rare earth neodymium-iron-boron (NdFeB) magnets represent a minimally invasive adjunctive instrument for the retrieval of ferromagnetic MFBs; however, procedurally granular, anatomically stratified data from dedicated hand surgery practices remain limited.

**Methods:**

A retrospective review of 15 consecutive patients who underwent neodymium magnet-assisted retrieval of upper extremity MFBs at a single institution between January 2022 and January 2024. N35-grade NdFeB disc magnets (10 × 1.5 mm; 1.17–1.20 Tesla) and a 36 mm fishing magnet were employed. Two operative techniques were applied: *direct removal*, in which the magnet attracts the MFB through a mini-incision; and *vibration localization*, in which magnet-induced fragment movement enables precise pre-extraction localization. Primary outcomes were procedural success, operative duration, and fluoroscopy utilization.

**Results:**

All 15 MFBs were successfully retrieved (100% procedural success). Mean operative duration was 3.13 ± 1.35 min overall; the hand cohort (*n* = 12) demonstrated a mean of 2.75 ± 1.14 min under local anesthesia. Mean fluoroscopy utilization was 0.88 images per case (total: 13 images). Twelve patients (80%) underwent superficial mini-incisions without deep dissection.

**Conclusion:**

In this preliminary descriptive series, neodymium magnet-assisted retrieval achieved 100% procedural success with low fluoroscopy utilization, brief operative durations, and uncomplicated short-term wound healing in all patients. These findings should be interpreted as hypothesis-generating given the limited sample size and support the feasibility—rather than the proven comparative superiority—of this approach within a dedicated hand surgery practice.

## 1 Introduction

Retained metallic foreign bodies (MFBs) constitute a recognized and clinically consequential subset of soft tissue injuries encountered across multiple surgical disciplines. Their etiology is diverse, encompassing industrial metalworking accidents; iatrogenic events—including fractured intraoperative needles and displaced acupuncture instruments; and non-powder firearm injuries such as those caused by ball-bearing (BB) guns (1–3). The potential sequelae of retained MFBs are substantial, ranging from chronic pain, wound infection, and osteomyelitis to reactive fibrosis and iatrogenic neurovascular injury during attempted retrieval—collectively underscoring the importance of safe and effective extraction strategies.

Conventional surgical retrieval is associated with well-documented procedural limitations: requirements for broad incisions, extensive tissue dissection to localize impalpable fragments, prolonged operative durations, and reliance on repeated intraoperative fluoroscopic imaging (3–5). These factors increase the risk of iatrogenic injury to adjacent neurovascular structures and impose a disproportionate burden on theater resources without guaranteeing complete retrieval (4, 5).

Rare earth neodymium-iron-boron (NdFeB) magnets have emerged as an adjunct retrieval instrument that may mitigate many of these limitations. By virtue of their exceptionally high magnetic flux density relative to their physical dimensions, NdFeB magnets can exert sufficient attractive force to draw ferromagnetic fragments through a minimal incision, circumventing the need for wide surgical exposure (2, 6, 7). The feasibility of this technique was first demonstrated by Chin et al. in 2000 (5) and subsequently corroborated by Dolderer et al. within a plastic surgery context (6). The most comprehensive published series—Xing et al.'s 7,390-case cohort—established a procedural success rate of 99.5% across a mixed anatomical distribution, providing foundational proof of concept (4). More recently, Lukish et al. applied the technique to pediatric extremity injuries, emphasizing the ALARA principle, and Liu et al. described 100% success in 22 consecutive adult limb cases with a mean fragment depth of 2.35 cm from the body surface (7, 8).

Despite this growing body of literature, anatomically stratified operative data from a dedicated hand surgery practice—where the proximity of digital neurovascular bundles, flexor tendon sheaths, and small joint capsules imposes specific technical constraints—remains infrequently reported. The objective of this study is to describe our institutional operative technique and document procedural parameters for neodymium magnet-assisted MFB retrieval across 15 consecutive upper extremity cases, providing procedurally specific, subgroup-stratified data of practical utility to hand surgeons considering adoption of this approach.

## 2 Materials and methods

### 2.1 Study design and ethical approval

This project is a single-institution retrospective descriptive case series of consecutive patients presenting between January 2022 and January 2024 with upper extremity MFBs requiring surgical intervention. The study was approved by the Başakşehir Çam and Sakura City Hospital Ethics Review Committee (Protocol No. 2024-240) was conducted in full accordance with the principles of the Declaration of Helsinki (revised 2013). As this was a retrospective review, no prospective study enrollment occurred; written informed consent for the use and publication of clinical data and intraoperative images was obtained from all participants at the time of data collection.

### 2.2 Patient selection

Inclusion criteria: (1) radiographically confirmed MFB in the upper extremity; (2) MFB confirmed or strongly suspected to be ferromagnetic based on injury mechanism and imaging characteristics; and (3) surgical retrieval performed using neodymium magnet assistance as the primary operative technique.

Exclusion criteria: (1) confirmed non-ferromagnetic MFB composition; (2) the primary procedure was formal wound debridement for gross contamination; or (3) incomplete operative records.

### 2.3 Instrumentation

Two magnet configurations were employed. The primary instrument was a commercially available NdFeB disc magnet (10 mm × 1.5 mm diameter; N35 grade; magnetic flux density 1.17–1.20 Tesla; coercive force > 860 kA/m). For cases requiring broader surface coverage or deeper fragment localization, a larger fishing magnet (36 mm diameter; N35 grade; identical flux density) was utilized. Both instruments were sterilized according to institutional protocol prior to intraoperative deployment.

### 2.4 Operative technique

**Figure 1:**
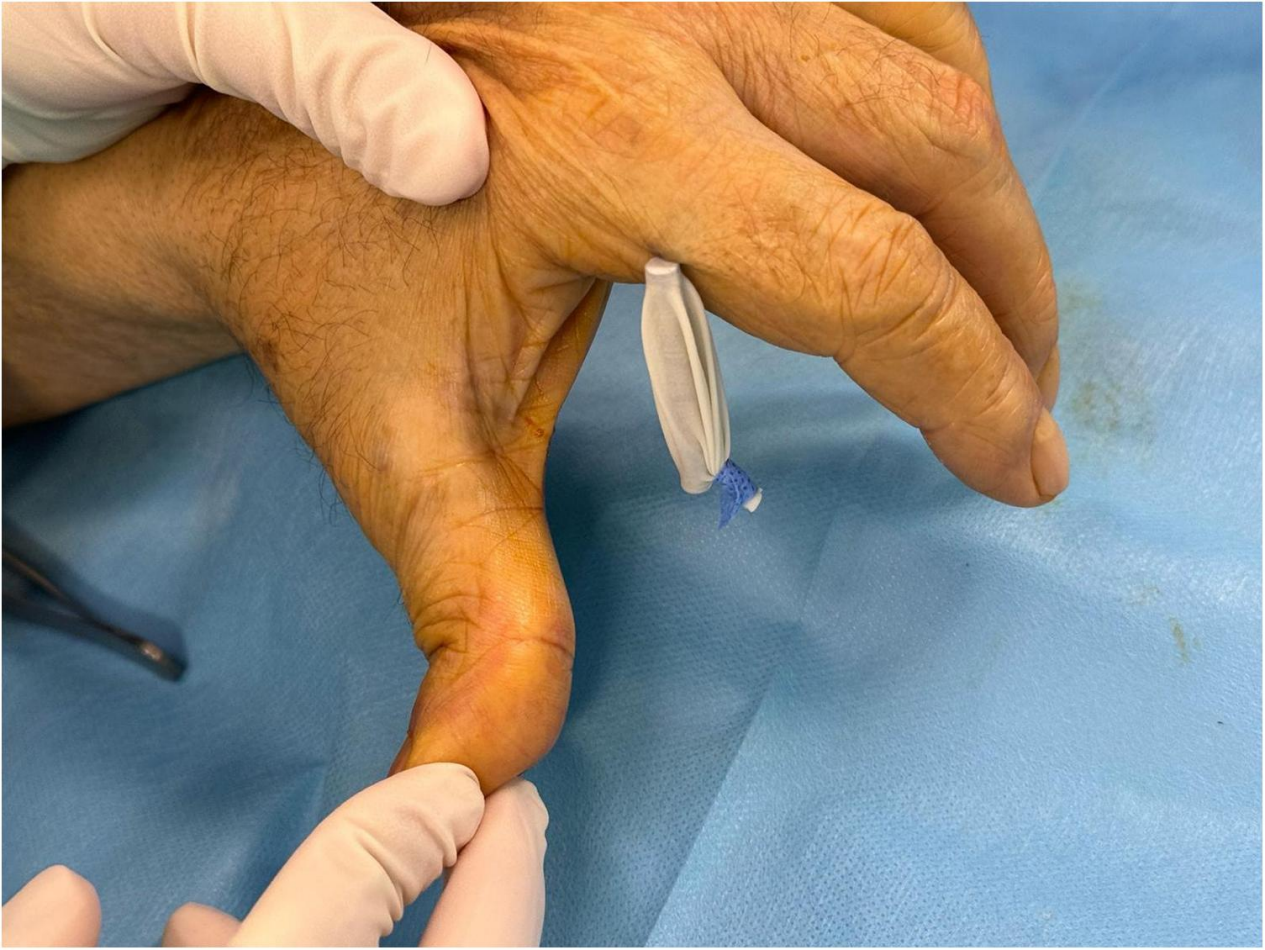
Intraoperative photograph demonstrating the *direct removal* technique. The sterilized N35-grade NdFeB disc magnet (10 mm × 1.5 mm) is positioned within the mini-incision, exerting direct magnetic attraction on the ferromagnetic fragment and drawing it to the instrument surface without requirement for broad tissue dissection or extensive wound exploration.

**Figure 2:**
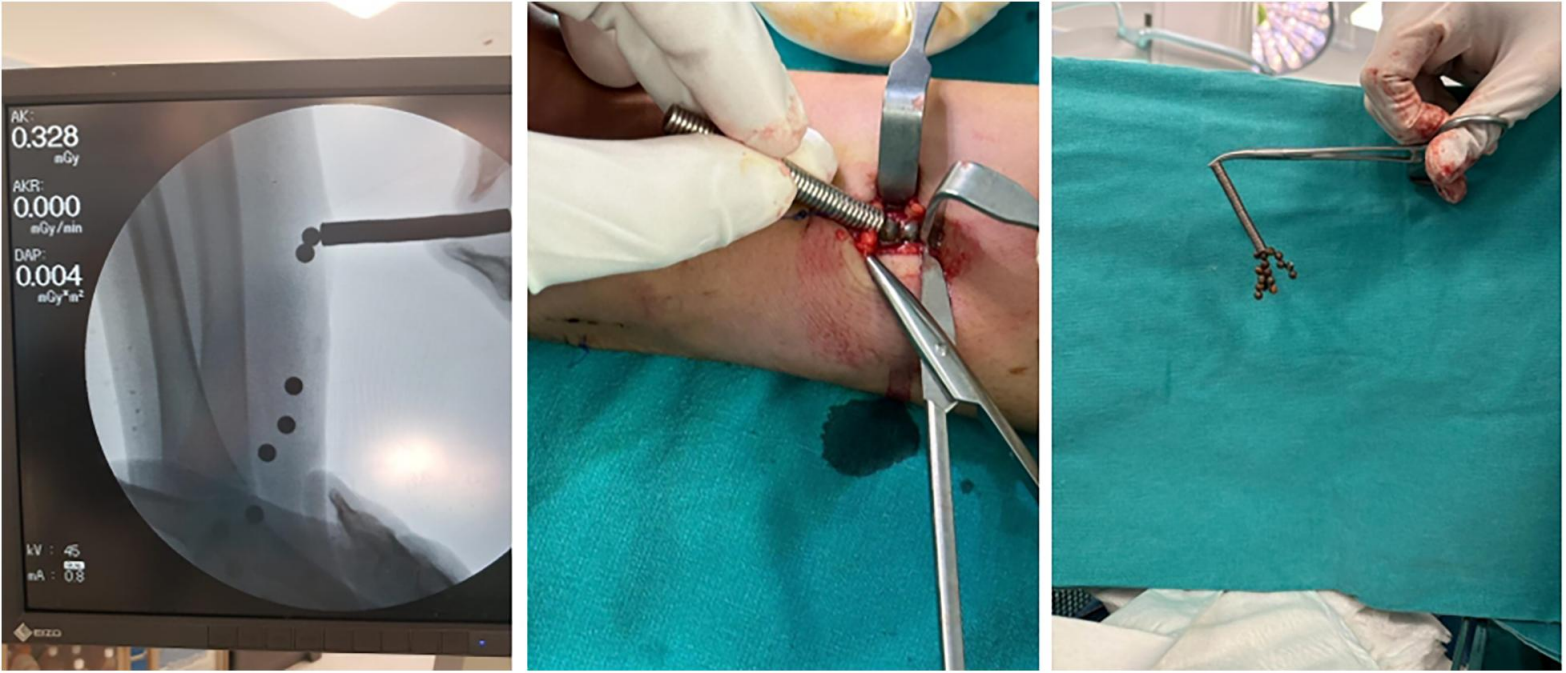
Illustrative diagram of the *vibration localization* technique. The disc magnet is swept systematically across the operative field; induced movement of the embedded metallic fragment generates a palpable vibratory sensation, enabling precise three-dimensional localization prior to targeted extraction with surgical forceps.

All procedures commenced with conventional plain radiographic assessment to confirm the location and approximate depth of the MFB. Following sterile preparation of the operative field, the sterilized magnet was applied to the intact skin surface to identify the point of maximal magnetic attraction—the anatomical locus at which the MFB lay in closest proximity to the dermis. A mini incision was fashioned at this site, sized to permit magnet insertion while minimizing soft tissue disruption. Two complementary retrieval techniques were employed selectively shown in Figures 1, 2:

Direct Removal: The magnet was introduced into or immediately adjacent to the incision. The magnetic field directly attracted the MFB, drawing it toward the instrument surface. Successful fragment capture was frequently confirmed by an audible or tactilely perceived contact between the fragment and magnet prior to withdrawal (8).
Vibration Localization: When direct capture was not immediately achieved, the magnet was swept systematically across the operative field. The induced movement of the MFB within the surrounding soft tissues generated a palpable vibratory sensation, enabling precise three-dimensional localization before targeted extraction with surgical forceps (8).

Intraoperative fluoroscopy was employed at the operating surgeon's discretion, principally to confirm initial fragment position or verify complete retrieval before wound closure. The choice of local versus general anesthesia was determined preoperatively based on anticipated dissection complexity and wound geometry. Technique selection between direct removal and vibration localization was governed prospectively at the time of surgery by wound geometry, fragment depth, and intraoperative tactile feedback, rather than by a predetermined algorithm. Magnet-assisted retrieval was applied only to fragments confirmed or strongly suspected to be ferromagnetic on preoperative imaging; in the hand specifically, magnet use was restricted to superficial zones remote from digital neurovascular bundles, flexor tendon sheaths, and small joint capsules, with fragment depth and trajectory characterized on preoperative imaging before any attempt at magnetic traction.

### 2.5 Outcome measures and statistical analysis

Primary outcomes were (1) procedural success, defined as complete radiographic and clinical confirmation of MFB removal; (2) operative duration, measured from skin incision to wound closure; and (3) intraoperative fluoroscopy utilization, expressed as the number of images per case. Secondary descriptive variables included anesthesia type, anatomical injury region, and depth of dissection required. Operative durations are presented as mean ± standard deviation (SD). This study was designed as a retrospective, descriptive case series rather than a comparative study; accordingly, no concurrent or historical control cohort was established, and no formal statistical inference analysis was performed**.**

### 2.6 Postoperative follow-Up

All patients were assessed at routine postoperative visits for wound healing, signs of infection, and residual sensory or motor symptoms in the operated limb. Follow-up duration is reported in Results.

## 3 Results

Fifteen patients underwent neodymium magnet-assisted retrieval of upper extremity MFBs during the study period. Complete retrieval was achieved in all 15 cases (100% procedural success rate). No intraoperative complications—including neurovascular injury, instrument failure, or wound contamination—were recorded in any patient. Clinical profiles, procedural characteristics, and operative outcomes are summarized in Table 1.

**Table 1:** Anatomical distribution, procedural characteristics, and operative outcomes of 15 consecutive patients treated with neodymium magnet-assisted upper extremity metallic foreign body retrieval. Operative duration values are expressed as mean ± standard deviation (SD). Fluoroscopy utilization represents the mean number of images acquired per patient within each anatomical subgroup.

Anatomical region	N	Anaesthesia	Injury type	Surgical approach	Mean fluoroscopy (images/case)	Op. duration (min, Mean ± SD)
Hand	12	Local	Small metallic fragments/fractured needles	Mini-incision; no deep dissection	0.6	2.75 ± 1.14
Forearm and wrist	2	Local	Small metallic fragments/fractured needles	Mini-incisions; no deep dissection	1.0	4.50 ± 1.54
Forearm, wrist, and hand	1	General	Gunshot wound; multiple spherical metallic bodies	Deep neurovascular bundle dissection	4.0	5.00
Total	15	—	—	—	0.88	3.13 ± 1.35

### 3.1 Anatomical distribution

The hand was the predominant anatomical site of injury (*n* = 12; 80%), followed by the forearm and wrist (*n* = 2; 13%), with one combined forearm, wrist, and hand case (*n* = 1; 7%) involving a high-velocity gunshot wound with multiple retained spherical metallic bodies. The majority of patients (*n* = 14; 93%) had sustained injuries from small ferromagnetic fragment penetration or iatrogenic needle fracture.

### 3.2 Anesthesia and surgical approach

Fourteen patients (93%) underwent retrieval under local anesthesia. Twelve of these (80%) required only a superficial mini-incision without deep dissection, confirming the minimally invasive character of the technique in appropriately selected cases. The single gunshot wound case necessitated general anesthesia and formal neurovascular bundle dissection, reflecting the complexity inherent to that injury pattern rather than a technique-specific limitation.

### 3.3 Operative duration and fluoroscopy utilization

The overall mean operative duration was 3.13 ± 1.35 min. The hand cohort demonstrated the shortest mean operative time at 2.75 ± 1.14 min, consistent with the superficial fragment depth and simpler wound geometry of that subgroup. Forearm and wrist cases required a mean of 4.50 ± 1.54 min, and the single complex gunshot wound case required 5.00 min. The mean intraoperative fluoroscopy utilization was 0.88 images per case (range: 0–4; total 13 images). Hand cases required the fewest fluoroscopic exposures, at a mean of 0.6 images per case. Selected published cases providing comparative clinical context are summarized in Table 2.

**Table 2:** Summary of selected PubMed-indexed published cases demonstrating neodymium magnet-assisted metallic foreign body retrieval across diverse clinical and anatomical contexts.

Patient case (reference)	MFB location	Key outcome
48-year-old blacksmith (1)	Cheek; impalpable fragment	Retrieved under local anaesthesia through entry wound; wide exploration avoided
13-year-old; BB gun injury (2)	Forehead	5 mm incision; local anaesthesia; excellent cosmetic result; no fluoroscopy
Paediatric extremity MFBs *n* = 11 (6)	Extremity soft tissue	100% retrieval; cross-sectional imaging avoided; median operative time 2.5 min
22 adult limb cases (7)	Upper and lower extremity	100% retrieval; mean fragment depth 2.35 cm from body surface

### 3.4 Postoperative outcomes

All 15 patients were followed clinically for a minimum of 6 months. Wound healing was uncomplicated in all cases, with no instances of surgical site infection, wound dehiscence, or fragment recurrence. No patient reported residual neurovascular symptoms attributable to the retrieval procedure at last follow-up.

## 4 Discussion

This descriptive series documents the procedural experience and operative outcomes of neodymium magnet-assisted ferromagnetic MFB retrieval in 15 consecutive upper extremity cases at a dedicated hand surgery unit. A 100% retrieval success rate was achieved across a clinically heterogeneous cohort spanning superficial digital needle injuries under local anesthesia to a complex multi-fragment gunshot wound requiring general anesthesia and deep neurovascular dissection. The majority of patients benefited from superficial mini-incisions with low fluoroscopy utilization and operative durations under five minutes.

These findings are consistent with the existing literature and extend it in an upper extremity-specific context. The earliest descriptions of rare earth magnet use for foreign body retrieval date to Chin et al. in 2000 (5) and Dolderer et al. in 2004 (6). Xing et al.'s 7,390-case series established a 99.5% overall procedural success rate across a mixed anatomical cohort (4). Lukish et al. reported the technique in pediatric extremity injuries (*n* = 11), emphasizing radiation minimization under the ALARA principle (7), and Liu et al. most recently reported 100% success in 22 adult limb cases, with a mean fragment depth of 2.35 cm from the body surface (8). Our series contributes anatomically stratified operative time and fluoroscopy data specifically from a hand surgery practice, a context in which these parameters have not previously been reported in disaggregated subgroup form. Direct comparison with Xing et al.'s 7,390-case series is informative despite the disparity in scale. Their cohort spanned a heterogeneous mix of anatomical sites with a 99.5% procedural success rate but did not report subgroup-specific operative duration or fluoroscopy data for the upper extremity nor distinguish hand-specific outcomes from the broader cohort (3). Our series, although markedly smaller, is the first to our knowledge to report these parameters disaggregated within a dedicated hand surgery practice, where proximity to neurovascular and tendinous structures imposes constraints not generalizable from a mixed-anatomy series. Fan et al.'s case report of a sewing needle retrieved from the sacrum using a permanent magnet further illustrates the technique's extension beyond the extremities, reinforcing that magnet-assisted retrieval is governed more by fragment ferromagnetism and surgical access than by anatomical site *per se* (9).

### 4.1 Technical considerations

Technique selection was governed by wound geometry, fragment depth, and intraoperative tactile feedback rather than a predetermined algorithm—consistent with the adaptive approach described in the broader literature (1, 3).

Rigorous preoperative patient selection is a prerequisite for safe technique application. Magnet-assisted retrieval applies exclusively to ferromagnetic MFBs; non-ferromagnetic materials—including aluminum, copper, and glass—will not respond to magnetic traction. Chronically retained fragments may develop a surrounding fibrous pseudocapsule mechanically impeding extraction, requiring preliminary capsulotomy. Horoshun et al., in an analysis of 623 magnetic instrument procedures, identified recurrent technical error categories directly relevant to patient selection and operative planning (10), summarized in Table 3. These documented error categories directly informed the preoperative assessment criteria applied in the present series.

**Table 3:** Recurrent technical error types, frequencies, and clinical implications identified in a systematic analysis of 623 surgical magnet procedures.

Error type	Error rate	Clinical implication
Use not as indicated	30.6%	Most frequent error. Requires pre-operative imaging confirmation and clear clinical indication based on injury mechanism.
Failure to use imaging guidance	18.4%	Proceeding without radiographic or ultrasound guidance to confirm fragment position prior to incision is a potentially dangerous practice.
Ignoring the fibrous capsule	16.3%	Chronically retained MFBs develop a fibrotic pseudocapsule resisting magnetic traction. Preliminary capsulotomy required before magnet retrieval can succeed.
Retrieval of non-ferromagnetic bodies	14.3%	Injury history and radiographic evaluation are mandatory to confirm iron-based MFB composition prior to magnetic retrieval attempts.
Disregarding the wound channel	11.2%	Failure to account for wound channel trajectory and angulation can obstruct magnet access and risk inadvertent collateral tissue injury.
Disregarding anatomical hazard zones	6.1%	Strong magnetic forces should not be applied near major neurovascular bundles; technique is safest in superficial zones remote from critical structures.

### 4.2 Hand surgery-specific considerations

The hand surgery context magnifies the consequences of the anatomical hazard zones described in Methods. Because digital neurovascular bundles, flexor tendon sheaths, and small joint capsules tolerate little margin for uncontrolled fragment acceleration, the low fluoroscopy utilization observed in our hand cohort (mean 0.6 images/case) is not merely a procedural efficiency but a meaningful reduction in cumulative radiation exposure in a population—often young, working-age patients with hand injuries—for whom repeated imaging carries longer-term relevance.

### 4.3 Strengths and limitations

#### 4.3.1 Strengths

This series provides procedurally granular, anatomically stratified descriptive data from a dedicated hand surgery practice—a context underrepresented in the existing literature. The 100% procedural success rate across a heterogeneous case mix supports the generalizability of the technique across a spectrum of upper extremity injury patterns. The explicit documentation of two operative technique variants with selection criteria provides a reproducible framework for adoption.

#### 4.3.2 Limitations

This research project is a retrospective, uncontrolled, single-institution descriptive study of 15 cases. No contemporaneous comparison cohort was available; accordingly, no inferences regarding comparative efficacy, operative time savings, or fluoroscopy reduction relative to standard methods can be drawn. The operative times and fluoroscopy counts reflect the combined effect of technique, individual surgeon experience, and specific case mix—all of which may differ in other practice settings. Patient-reported outcomes and longer-term clinical endpoints were not systematically captured. The success rate must be interpreted within the statistical constraints imposed by a small sample. One reference cited for technical error analysis [Horoshun et al., reference (10)] is not indexed on PubMed; authors are advised to verify or replace this source as required by the target journal prior to final submission.

Prospective comparative studies—including matched cohort designs or randomized allocation of magnet-assisted vs. conventional retrieval in appropriate patients—are required to rigorously quantify the clinical and health-economic advantages of this technique in upper extremity surgery.

## 5 Conclusion

In this descriptive series of 15 patients, neodymium magnet-assisted retrieval of upper extremity ferromagnetic MFBs achieved a 100% procedural success rate with brief operative durations and low intraoperative fluoroscopy utilization in the majority of cases. Two complementary operative techniques—direct removal and vibration localization—provided adaptable retrieval modalities across a range of wound geometries and fragment characteristics. These results are consistent with prior published experiences and support the procedural feasibility of this approach within a dedicated hand surgery practice. Formalized prospective comparative studies are necessary to define the comparative advantages of neodymium magnet-assisted retrieval over conventional methods with respect to operative efficiency, radiation exposure, and patient-centered outcomes in the upper extremity.

## Data Availability

The original contributions presented in the study are included in the article/Supplementary Material, further inquiries can be directed to the corresponding author/s.

## Supplementary material

Supplementary Video 1 Fishing technique.
